# Cultural competence training of dieticians: development and preliminary evaluation

**DOI:** 10.1017/S1463423624000483

**Published:** 2024-10-28

**Authors:** Mirjam Jager, Susanne Leij-Halfwerk, Reinier Akkermans, Rob van der Sande, Maria van den Muijsenbergh

**Affiliations:** 1 Nutrition and Dietetics, HAN University of Applied Sciences, Nijmegen, The Netherlands; 2 Department of Primary and Community Care, HAN University of Applied Sciences, Nijmegen, The Netherlands; 3 Department of Primary and Community Care, Radboud Institute for Health Sciences, Radboud University Medical Center, Nijmegen, The Netherlands; 4 Diakonessenhuis, Utrecht, The Netherlands; 5 Radboud University Medical Centre, Radboud Institute for Health Sciences, IQ healthcare, Nijmegen, The Netherlands

**Keywords:** Cultural competence, dietetic care, dieticians, ethnic minorities, migrants, observational assessment

## Abstract

**Introduction:**

Training can improve healthcare providers’ cultural competence and increase their awareness of bias and discrimination in medical decision-making. Cultural competences training is lacking in the education of dieticians in the Netherlands. The aim of this study was to describe the pilot-implementation of a cultural competence training for dieticians and preliminary evaluation of the training.

**Methods:**

A training was developed based on Seeleman’s cultural competence framework and previously held interviews with migrants, dieticians, and experts. The training consisted of a mixture of didactic and experiential methods, alternating knowledge transfer with exercises to increase awareness, reflection, and feed-back on recorded consultations, and communication training with migrant training actors. The training was piloted in 8 participating dieticians and preliminary mixed-method evaluation was done using a Cultural Competence Questionnaire, Experience Evaluation Questionnaire, and consultation observations.

**Results:**

The questionnaires showed that dieticians were positive about the training. They found it valuable and educational. Participants reported an increase in self-perceived cultural competence and attitudes. Knowledge and skills remained approximately the same. The observations showed that dieticians applied the teach-back method and discussed treatment options more often after training. There was no increase in the use of visual materials.

**Conclusion:**

The training was well appreciated and, although a small-scale pilot, this mixed-method study suggests an ability to change cultural competence. The combination of a self-assessment instrument and consultation observations to evaluate cultural competence was highly valuable and feasible. These encouraging results justify a broader implementation of the training.

## Introduction

In ethnic minority populations, health in general is worse compared to the ethnic majority population (Rechel *et al.*, 2013). This is also the case in the Netherlands (CBS, 2020). Type 2 diabetes mellitus (DM2) for example is two to four times more prevalent in ethnic minority populations compared to native inhabitants (Meeks *et al.*, 2016). Other (chronic) diseases such as asthma, dementia, coronary heart diseases, anxiety disorders, and stroke are more prevalent in ethnic minorities compared to the Dutch ethnical majority (Kunst *et al.*, 2008; Parlevliet *et al.*, 2016) and both quality and outcomes of healthcare are worse. Diabetes care, for example, is of lower quality and less effective in ethnic minorities, leading to higher rates of complications and higher healthcare costs (Lanting *et al.*, 2008).

To deliver effective care for migrant patients of different ethnic, socio-cultural, and linguistic backgrounds, dieticians must team well with these patients to obtain information, discuss treatment options, and to establish motivation and skills for self-management. To enable dieticians to do this, they have to possess cultural competences that are defined by Seeleman (Seeleman *et al.*, 2009): *knowledge* about ethnic differences in morbidity and treatment, *awareness* of how culture shapes individual behaviour, social contexts and one’s own prejudices and *skills* to transfer information in a way the patient can understand, to know when external help with communication is needed, and to adapt to new situations creatively. Embedding these competences in our healthcare system and the training of dieticians enables the provision of appropriate, patient-centred care to patients with diverse values, beliefs, and behaviours (Horvat *et al.*, 2014).

Patient centred care improves patient-professional relationship, patient and clinician satisfaction, as well as healthcare outcomes like greater adherence to treatment and improved quality of life at lower healthcare costs (De Silva, 2014; De Boer *et al.*, 2013; Stewart *et al.*, 2000; Saha and Beach, 2011; Olsson *et al.*, 2013; Altin and Stock, 2016; Kinmonth *et al.*, 1998). Our previous studies (Jager *et al.*, 2018, 2020) showed that both dieticians and patients were struggling with delivering and receiving patient centred dietetic care. Migrant patients often experienced difficulties within their community when adhering to dietary advice (Jager *et al.*, 2018; Kohinor *et al.*, 2011) and often expect a more rigorous, directive approach of dietetic advice, based on medical tests (Jager *et al.*, 2018). Dieticians experienced difficulties in promoting effective self-management and adherence to dietary advice in migrant patients (Jager *et al.*, 2020; Fransen *et al.*, 2015). Main barriers they experienced were language differences, a lack of knowledge of cultural differences in eating behaviour, and insufficient awareness of their patients’ health literacy. Furthermore, they expressed a desire for more training in providing care for migrants (Jager *et al.*, 2020). In our observational study of dietetic consultations we found that formal interpreters were never used and the patient’s literacy, nor the patient’s understanding of the consultation were checked during consultations. The food intake assessment was often incomplete and visual materials were used infrequently (Jager *et al.*, 2023). In conclusion, our studies underpinned the desirability of training for dieticians in cultural competences.

Although it is not yet clear whether cultural competence training improves patient outcomes (Lie *et al.*, 2011; Renzaho *et al.*, 2013; Truong *et al.*, 2014), reviews have shown that training can improve healthcare providers’ cultural competence and increase their awareness of bias and discrimination in medical decision-making (Renzaho *et al.*, 2013; Horvat *et al.*, 2014; Govere and Govere, 2016). In several countries, cultural competence training is mandatory (Dogra *et al.*, 2010). Yet, a recent study showed that there are major deficiencies in the implementation of cultural competence training within European medical curricula (Sorensen *et al.*, 2019). This is also the case in the education of dieticians in the Netherlands where cultural competences training is lacking.

Therefore, we developed a post-graduate training in culturally competent, person-centred dietetic care, tailored to the needs of dieticians and migrant patients (which is described below in the methods section). To assess whether this training format could be able to modify cultural competence behaviour in dieticians, we developed a combination of evaluation instruments. If these seem promising, the training might be developed further and be implemented in larger cohorts.

The aim of this study was to describe the pilot-implementation of a cultural competence training for dieticians and preliminary evaluation of the training.

## Methods

The study was approved by the Medical Ethical Committee of the Central Committee on Research involving Human Subjects region Arnhem and Nijmegen (reference 2014/210).

### Development of the training

#### Theoretical Framework

**Figure 1.**
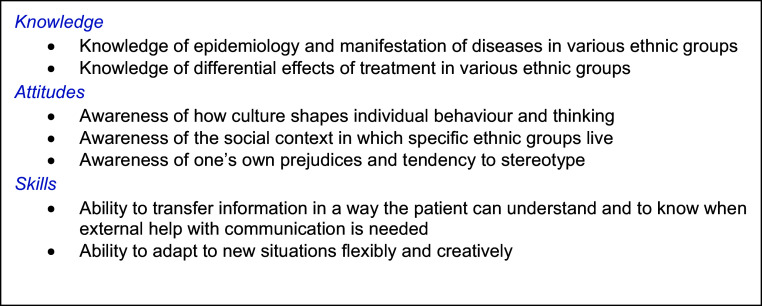
Seeleman’s framework of cultural competence (Seeleman et al., 2009).

The general outline of the training was based on Seeleman’s cultural competence framework, see Figure 1 (Seeleman *et al.*, 2009) and studies into effective transfer of knowledge and skills into daily practice, like working with an interpreter, assessing literacy levels, and checking mutual understanding (Ha Dinh *et al.*, 2016).

#### Tailoring the training to the needs of migrant patients and dieticians

To tailor the content of training to the requirements of migrant patients and dieticians, we re-examined previously held semi-structured interviews with 12 migrant patients from Turkey, Morocco, Iraq, and Curacao, who visited a dietician (Jager *et al.*, 2018) as with 12 dieticians of various age, ethnic background and working experience (Jager *et al.*, 2020). In addition, we interviewed ten experts in culturally competent healthcare, cultural competence training and training of dieticians. We collected information on the three pillars of Seeleman’s framework: desirable attitude, knowledge, and skills of dieticians, and on learning needs as well as optimal didactic forms. Box 1 summarizes the three interview studies. In Table 1, the results of these studies are translated into desirable content of the training, related to knowledge, attitude, or skills of dieticians as well as optimal didactic forms.

Box 1. Summary of interview studies with perspectives of patients, dieticians, and expert opinion

**Table:**
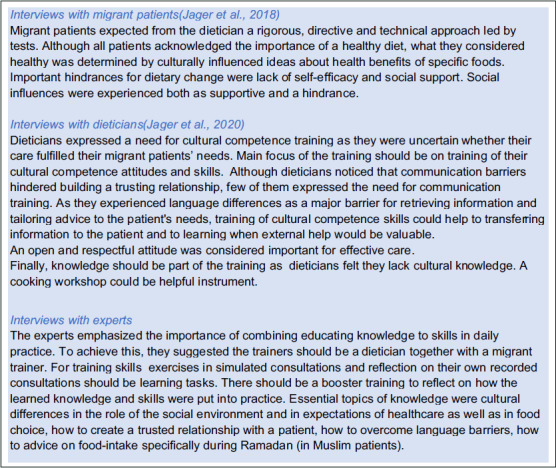

**Table 1.** Theoretical framework of the content and format of the training based on theory and three perspectives

*Knowledge*	• Knowledge of cultural differences in food choice and preparation • Knowledge of implications of Ramadan • Knowledge of the prevalence of language barriers and low (health) literacy • Knowledge of the importance of family and social influence • Knowledge of when and how to involve a professional interpreter in the consultation
*Attitudes*	• Awareness of the possibly culturally influenced expectations of patients • Awareness of the social context that influences eating and possibilities to adhere to diet recommendations • Awareness of one’s own ideas about good conduct during consultations • A curious and flexible attitude
*Skills*	• Ability to foster self-efficacy • Ability to build a trusting relationship • Ability to apply different modes of conduct (e.g., more or less directive) • Ability to communicate in easy to understand language and to use visual support in diet intake assessment and diet and health promotion • Ability to assess literacy levels and to check mutual understanding patient (teach-back) • Ability to communicate across language barriers e.g., how to involve an interpreter • Ability to adapt to new situations flexibly and creatively
*Format of the training*	• Involve migrant trainer as well as dietician trainers • Cooking workshop • Include practical communication skills training • Include observation of consultations • Include booster training

#### The final training

The final training consisted of a mixture of didactic and experiential methods, alternating knowledge transfer with exercises to increase awareness, reflection, and feed-back on video/audio recorded consultations of the participants, and practice skills sessions in which participants performed consultations with migrant training actors (Table 2).

**Table 2.** Final training layout: components of the cultural competence training

Training component	Specification
*Day 1*	
Awareness exercise	Exercise to increase awareness of one’s own culture (attitude).
Cooking workshop	Led by migrant dietician. Participants cooked and tasted several Turkish, Moroccan, and Surinam dishes and discussed nutritional aspects (knowledge and skills).
Intercultural communication lecture and communication training	Led by intercultural communication dietetics teacher and migrant intercultural communication trainer/actor. Addressed topics such as: differences in expectations of healthcare, use of interpreter, discussing treatment options, social environment. Participants had simulated conversations about topics they found challenging (knowledge and skills).
*Day 2*	
Interactive lecture on low literacy with communication training	Lecture led by expert, together with an experience expert. Topics addressed were: awareness of low literacy, recognizing and adapting the consultation, visual materials, teach back method, simulated consultations to practice (knowledge, attitude, and skills)
Lecture and on food cultures and food intake anamnesis	Led by migrant dietician. Topics addressed: food cultures in the Netherlands, awareness of differences within cultures, assessment of preparation methods, portion sizes, and use of visual tools (knowledge, attitude, and skills).
Small group discussion of recorded consultations	In small groups, participants discussed their recorded consultation, exchange of tips and feedback (attitude, skills).
Lecture on diabetes during Ramadan	Topics addressed: knowledge on nutrition and medication during Ramadan, practicing dietetic advice during Ramadan (knowledge and skills)
Kahoot quiz	Quiz to refresh the leaned knowledge during day 1 and 2 (knowledge)
*Day 3 Booster Session*	
Exercise culture and manners	Led by intercultural communication expert. Exercise and discussion about cultural differences in manners and communication (attitude).
Case discussion	Participants worked on a given case and discussed their approaches (skills)
Refreshment and summary of content of the training	All key topics were summarized and lessons learned were discussed (knowledge).
*Additional assignments*	
3 recorded consultations	Participants recorded 3 consultations and handed in a written reflection. Trainers provided individual feedback on all consultations (attitude, skills)

### Evaluation instruments to assess the effect of the training

Cultural competence trainings are usually evaluated through self-assessment questionnaires only. However, as we aimed to assess actual behaviour as well, mixed methods were applied to assess the cultural competence of dieticians before, during, and after the pilot training. Cultural competence was assessed using a cultural competence questionnaire (CCQ) adapted from Seeleman (Seeleman *et al.*, 2014) and analyzing behaviour through recorded consult observations with migrant patients during the training. Experience was assessed by an experience evaluation questionnaire (EEQ). These instruments are described in more detail below.

#### Study population

Practising dieticians in the Netherlands were invited by announcements of the training on the websites of the Dutch Dietetic Association, the post-graduate education of the HAN University of Applied sciences and via LinkedIn. Also dieticians that participated in our previous studies (Jager *et al.*, 2018, 2020) were invited. Eight dieticians responded and were included in the pilot training. Written informed consent was obtained from all participants.

#### Assessment instruments

##### Self-assessment with CCQ

For the self-assessment of culturally competent knowledge, attitude, and skills, an existing questionnaire that was based upon the conceptual framework of Seeleman et al. (Seeleman *et al.*, 2009) was adjusted to the practice of dietetic care. Each domain of cultural competence was operationalized into questionnaire domains: i) General knowledge of ethnic minority care provision, ii) Reflection ability (attitude) for insight into one’s own understanding of prejudice and cultural frames of reference determined by the Groningen Reflection Ability Scale (GRAS) (Aukes *et al.*, 2007), and iii) Self-assessed cultural competent consultation behaviour (skills) during dietetic consultations with ethnic minority patients.

For general knowledge, eight multiple choice questions containing 42 items on minority care provision were used. The score was calculated as the sum of correct answers (‘correct’ = 1 point, ‘not-correct’, and ‘do not know’ = 0 points; care provision range 0–42. Reflection ability (GRAS) was determined by scoring the total of 10 statements that were rated to the level of agreement on a four-point Likert scale (1 = totally disagree, 4 = totally agree). To self-assess cultural competent consulting behaviour participants had to report on their own behaviour during consultation on: a) usings interpreters (maximum score 0–5), b) an 19-item scale on social context (maximum score 0–19), and c) 6 items about the use of communication techniques (maximum score 0–6). Points were given for cultural competent answers only (i.e., ‘correct’ = 1 point, ‘not-correct’, and ‘do not know’ = 0 points). For reasons of comparability, scores of all domain dimensions were also presented as percentage of the maximum possible score (Seeleman *et al.*, 2014). The original items on knowledge were updated to the current state of the art and the short case scenarios were adapted to match with the dietician’s profession. Identifying questions – i.e., on ethnic background – were removed to assure anonymity of the participants. Finally, self-perceived cultural competence (SPCC) was assessed using a 1–10 scale. Cultural competence in this item was described as ‘the knowledge, attitudes and skills required to provide adequate healthcare to patients of non-Dutch background’ (Seeleman *et al.*, 2014).

##### Consult observations

To evaluate the actual (changes in) behaviour during consultations, the participants recorded observations of their consultations before, during, and after the training that were scored with a cultural competence observation instrument that was developed previously.

This instrument was developed based on a systematic literature search (Jager *et al.*, 2020) and qualitative studies among migrant patients (Jager *et al.*, 2018) and their dieticians (Jager *et al.*, 2020). The development and face and content validity of the instrument were previously described (Jager *et al.*, 2023).

Behaviours were labelled on a 3-point scale as ‘well done, partly done, not/poorly done’, or not applicable’ for each relevant situation during the consultation. Done was labelled when a dietician showed the described behaviour partly or sufficiently. Not/poorly done was labelled when the behaviour was absent or performed in an insufficient manner. The option ‘not applicable’ was used in situations where the behaviour was not relevant or not possible.

All observations were scored by MJ and two senior year dietetic students independently. Scores were compared and discussed until consensus was reached.

##### EEQ

All participants were asked to complete a questionnaire twice to evaluate the appreciation of the training components, the trainers, report on their general impression of the training and lessons learned and provide tips to improve the training. The appreciation of each of the training components and trainers was rated on a scale of 1–10. A general impression, lessons learned and tips to improve the training were asked with an open question.

#### Data collection

**Figure 2.**
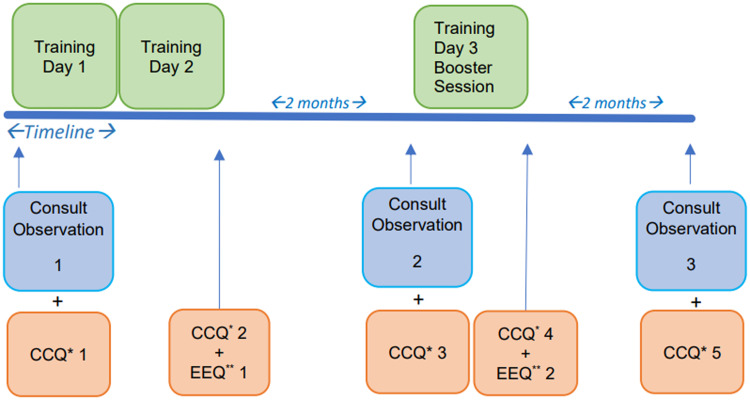
Cultural competence pilot training data collection.

Data were collected before the training day 1, after training day 2, and before and after day 3 (booster session) (see Figure 2).

#### Data analysis

##### CCQ

Results were analyzed using SPSS version 25. Participants’ characteristics were described by frequencies. As data were not normally distributed, median, and interquartile ranges were calculated for cultural competence domain scores and answers to separate questions.

Missing data were not imputed. Answers were checked for correct interpretation and excluded incorrect answers for analysis. Overall domain scores were calculated if no more than two subdomain items were missing. Because of the low number of participants, we did not test whether differences were significant.

##### Recorded observations of consultations

Regarding the observations, the number of consultations in which the behaviour was observed or not observed was counted for each behaviour. The results are presented combined for culturally competent behaviours and patient-centred care behaviours. For the purpose of brevity, as the observation instrument is elaborate, only behaviours that were clearly addressed during the training were reported.

##### EEQ

Results of the evaluation questionnaire (appreciation) were analyzed using descriptive statistics. Results from the open questions were summarized.

## Results

### Participants

Eight experienced dieticians participated in the pilot training (see characteristics Table 3). All were female. The majority had over 10 years of experience working as a dietician and all but one had 6 or more years working experience with migrant patients. Three had a migrant background: one Belgian, one Swedish, and one Armenian.

**Table 3.** Characteristics of dieticians participating in the training

Participant	Gender	Graduation year	Working experience as a dietician (years)	Years working with migrant patients	Ethnicity
1	Female	2000	>10 jaar	>10 jaar	Dutch
2	Female	1983	>10 jaar	6–10 jaar	Dutch
3	Female	1983	>10 jaar	1–5 jaar	Belgian
4	Female	2001	>10 jaar	>10 jaar	Dutch
5	Female	2011	6–10 jaar	6–10 jaar	Swedish
6	Female	2004	>10 jaar	>10 jaar	Dutch
7	Female	2005	>10 jaar	>10 jaar	Dutch
8	Female	2009	6–10 jaar	6–10 jaar	Armenian

### CCQ

**Figure 3.**
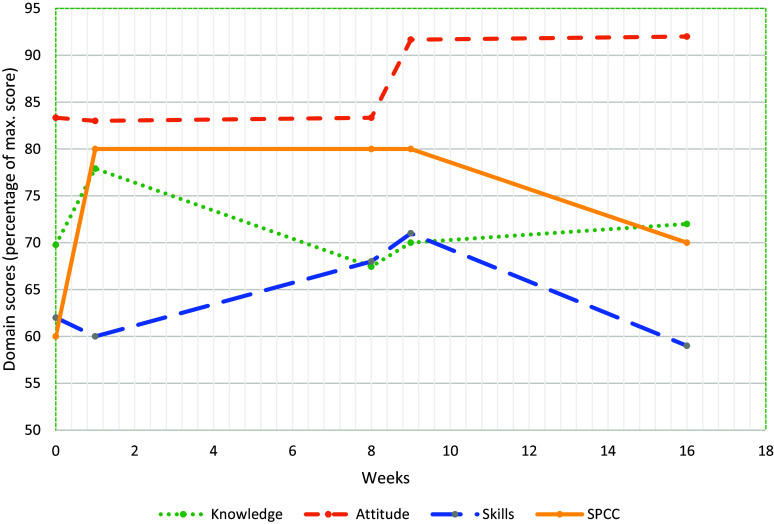
Median domain scores for knowledge, attitude, skills, and SPCC of dieticians at assessment times T1–T5 (*n* = 8).

**Figure 4.**
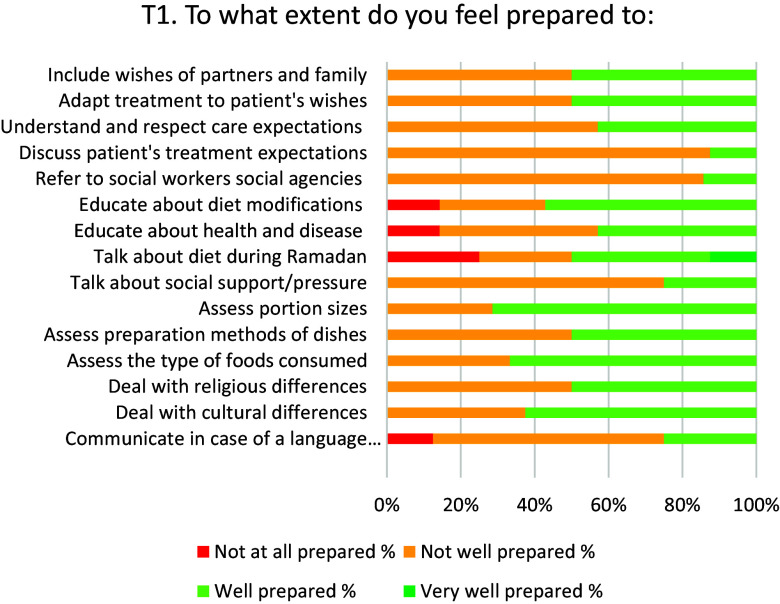
Aspects of self-perceived cultural competence at T1 (before training), in % of participants.

**Figure 5.**
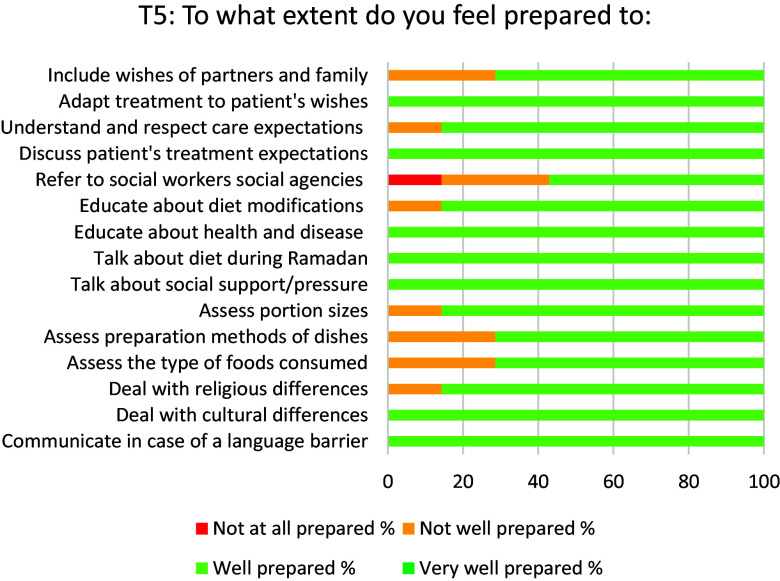
Aspects of self-perceived cultural competence at T5 (2 months after booster training), in % of participants.

The median domain scores for *knowledge* before training (T1) was 70% of the maximum score (see Table 4) and 72% two months after training (T5). In Figure 3, a slight increase in knowledge scores can be seen to 78% at T2, followed by a drop at T3–T5. Median domain scores for self-assessed *attitude* were 83% of the maximum score before training and increased to 92% after training. Median self-assessed skills domain scores were 62% before and 59% after the training. SPCC initially increased from 60% at T1 to 80% at T2, and dropped to 70% two months after the training. All aspects of SPCC improved after training (see Figures 4 and 5). For example, two months after training (T5) all participants felt well prepared for communicating in case of a language barrier. Furthermore, all participants felt well prepared to discuss treatment expectations with the patients.

**Table 4.** Median domain scores of the cultural competence (knowledge, attitude, skills) and SPCC* before and after training of dieticians (*n* = 8)

Domain	Before training (T1) median domain score in % of total score (IQR**)	2 months after training (T5) median domain score in % of total score (IQR**)	Difference before – after training (T1–T5) in % of total score
Knowledge	70% (17%)	72% (26%)	+2%
Attitude	83% (15%)	92% (8%)	+9%
Skills	62% (35%)	59% (24%)	−3%
SPCC*	60% (16%)	70% (10%)	+10%

* SPCC = Self-perceived cultural competence.

** = Inter-quartile range.

### Consult observations - Culturally competent behaviour assessment

In this paragraph, the results of the consultation observations are presented.

After the training, checking understanding via the teach back method had improved: it was observed in 3 out of 7 consultations, whereas before training this was never observed (See Table 5). Also negotiating about possible treatment options was done more often after the training as was providing the patient the opportunity to ask questions.

**Table 5.** Observed consultation behaviours, presented in number of consultations in which the behaviour was observed (*n* = 7 consultations)

Topic in the consultation	Behaviours	Before training (T1)		Two months after training (T5)	
		*Number of consultations*			
		Not/poorly done	Done	Not/poorly done	Done
Reason of the consultation	Explore the reason for the consultation, wishes and expectations*	0	2	0	2
Food intake assessment	Ask how meals are prepared*	1	1	0	2
	Assess time of meals*	0	2	0	2
	Assess what is consumed during each meal*	0	2	0	2
	Assess portion sizes*	0	2	0	2
Diagnosis	Check patient’s knowledge about diagnosis*	0	2	0	2
Treatment	Negotiate about possible treatment options	3	4	1	6
Communication	Check understanding via teach-back method	7	0	4	3
	Providing possibility and stimulating to ask questions	3	4	2	5
	Communicating via an interpreter*	0	0	0	2
	Use visual material during food intake assessment*	2	2	2	3
	Use visual material during diet recommendations*	3	2	2	2
	Use visual material during diet and health education*	1	0	2	0

* This behaviour was not applicable in all consultations, therefore the scores do not add up to 7.

Other behaviours, such as using visual materials during food intake assessment, were not observed more often after the training.

### EEQ

Participants assessed the cultural competence training as interesting and helpful. The average overall rating of the training is 7.9 out of 10.

Participants regarded the training as intensive, enriching, practical, varied, fun and repetition was appreciated. Participants most valued learning about adapting to patients with low literacy by keeping information simple, setting small goals at a time, taking on a slower pace and dosing the information. They also mentioned the importance of using the teach back method and learned to recognize and talk about low literacy. Participants valued acquiring knowledge about cultural differences in consumed products. Additionally, they mentioned they had more interest in other cultures and the context of their patients and recognize the importance of a trusting relationship. Finally, it was mentioned that they learned about cultural differences in care expectations.

## Discussion

A first cultural competence training for dieticians was developed and evaluated using a mixed-methods approach. The combination of a self-assessment instruments and consultation observation to evaluate cultural competence was highly valuable and feasible. The training was well appreciated and, although a small-scale pilot, this mixed-method study showed the ability to change cultural competence. Both the 2 questionnaires (CCQ and EEQ) and the observations seem to indicate that the training has a positive effect on self-perceived cultural competence and attitude. Skills and knowledge remained approximately the same. Observations showed that after the training the teach-back method and negotiating about possible treatment options were more often performed.

### Comparison with existing literature

The results of this small pilot study are encouraging. Self-perceived cultural competence increased after the training. Our study showed that after training, dieticians felt more prepared to discuss treatment expectations, adapt their treatment to the patient’s wishes, and communicate in case of a language barrier. This is hopeful, as our previous qualitative study showed that dieticians feel very uncertain when caring for migrant patients due to communication difficulties and experience a lack of ‘cultural’ knowledge. Other studies also found that training can diminish this uncertainty (Beach *et al.*, 2005).

The mixed methods approach we chose to evaluate the training is highly valuable. The combination of a CCQ and consultation observations provides a thorough insight into the learning outcomes of participants. It can even be argued that observation is an essential part of evaluation when training is aimed at changing consultation behaviour of any health professional. It is argued that direct observation can provide a more detailed, objective insight into what actually happens during a consultation (Seeleman *et al.*, 2012). This is especially relevant when socially desirable behaviours, such as culturally competent behaviours, are evaluated. Self-assessed cultural competence correlates with social desirability (Larson and Bradshaw, 2017) and correlates poorly with observer-rated cultural competence (Worthington *et al.*, 2000). This was also observed in our study. Results from the questionnaires regarding self-assessed use of the teach-back method did not correspond to observed use. For example before training, almost all participants indicated they use the teach-back method in practice. However, the observations showed that none of the participants used this technique before training. Another benefit of consultation observations is that the reflection of participants on their own consultations is valuable to stimulate awareness of one’s own blind spots. The only possible disadvantage is that observing and analyzing the recordings is labour intensive and requires training of the observants. However, the valuable information of observation far outweighs the cost of this investment and should be taken into account into training modules or research methodology.

The observation that participants incorporated the teach-back method more often into their consultations is encouraging, as this technique was not observed in consultations before training at all. Although there is still room for improvement, it is interesting that changes are observed within two to four months of training in this pilot sample. As teach-back is proven to be effective across a wide range of settings, populations and outcome measures (Talevski *et al.*, 2020), any increase in this behaviour of dieticians into their consultations is relevant.

Not all behaviours that were addressed in the training improved. For example, no increase in the use of visual materials was observed. This is unfortunate, as appropriate visual material use is important for conveying information to patients with low literacy (Sheridan *et al.*, 2011) and can help in case of a language barrier. This highlights the importance of paying substantial attention in the training to putting the learned knowledge, attitudes, and skills into practice and maintaining them. However, we recognize the complexities of cultural competence, and that training to change culturally competent behaviours into practice are non-linear and challenging (Dombrowski *et al.*, 2016). Experienced practitioners have routines that have to be changed. Fortunately, we know from the results of the questionnaire’s that participants feel well prepared to perform many of the behaviours taught in the training. However, it could be that learning a behaviour such as using visual materials remained at the stage of surface or deep learning as described in a model and review by Hattie and Donoghue (Hattie and Donoghue, 2016). They describe how learners progress from surface learning, through deep learning, to ultimately transfer. Transfer involves skills to transfer knowledge and understanding from one situation to a new situation. This is a dynamic process that requires learners to actively choose and evaluate strategies, consider resources, and to receive or seek feedback to enhance these adaptive skills. Strategies such as goal-setting, self-evaluation, and seeking peer-feedback can help to progress through the learning phases and may receive even more attention in our training. Most of these learning strategies, e.g., self-evaluation, goal setting, are used in our training. The use of peer-groups should be expanded, as we now only include trainer feedback on the recorded consultations. Small groups that discuss their learning goals, use role-play, discuss difficult situations, and provide each other with feedback could be a promising addition to a future training (Berkhof *et al.*, 2011; van der Vleuten *et al.*, 2019).

Implementation of cultural competence training into the bachelor educational program for dieticians is highly recommended. The teaching strategies used in this post-graduate training can easily be adapted to the bachelor program. Ensuring that dieticians are trained in cultural competencies before they enter the workforce prevents the feeling of uncertainty that was described in our previous studies (Jager *et al.*, 2020) and can help them to build effective routines.

Interestingly, knowledge and self-perceived cultural competence increased after the first two training days, yet declined over time. This result can be explained by the forgetting curve by Ebbinghaus. His theory explains how knowledge declines over time and explains why repetition is needed (Ebbinghaus, 2013). A booster training is therefore essential to maintain knowledge (Miller *et al.*, 2014). It may also be beneficial to send reminders of the main knowledge aspects to participants of trainings, e.g. by a 6-month reminder, via newsletters, or short video-lectures. SPCC did not decline until two months after training. This might be due to a loss of support after completing the training. Possibly, professional inter-vision, or connecting to a network of dieticians specialized in culturally competent dietetic care would be beneficial to provide continued support.

### Strengths and limitations

To our knowledge, this is the first cultural competence training for dieticians and description of a combination of evaluation instruments to assess the cultural competences in dieticians. We developed the training on a firm base of interviews with migrant patients, dieticians and experts and observations of consultations in real practice. Therefore, the training is based on the needs and wishes of the participants and we chose suitable didactical approaches.

Some of the participants in this pilot study are dieticians that were included in our previous studies into the cultural competence of dieticians. These participants may be more aware of and sensitive to culturally competent dietetic care. This could explain the high baseline results for attitudes from our CCQ and thus leave little room for improvement during the training. Since our goal was to describe the pilot-implementation of a cultural competence training for dieticians and preliminary evaluation of the training, we had a small sample size and did not strive for a representative sample yet. Therefore we cannot draw any conclusions on effect sizes. However, we do find the results encouraging and feel a broader implementation of the training is justified. Including larger and representative sample sizes will provide an opportunity to study the effects on cultural competence of dieticians.

Content validity of the assessment instruments should be assessed in a content validity study by systematically asking patients and professionals (e.g. dieticians, researchers) about the relevance, comprehensiveness and comprehensibility of the items, as recommended by COSMIN guidelines (Terwee *et al.*, 2017). Furthermore, interrater reliability should be studied, to assess the degree to which raters score consultations similarly.

### Recommendations for practice, education, and research

Since the evaluation of the pilot training yielded promising results, the training can be implemented on a larger scale. In addition, implementation of practical skills into daily practice may receive more attention and peer-groups should be formed during and after the training to discuss goals and difficult situations and provide each other with feedback.

Our pilot study aimed at exploring the feasibility of implementing and evaluating a cultural competence training. Our findings suggest that cultural competence training is promising. Cultural competence trainings should be evaluated using a combination of self-assessment questionnaires and consultation observation, as this provides essential information regarding both the learned knowledge, attitude, and skills and insight into the behaviour in real practice.
